# Mouse aversion to induction with isoflurane using the drop method

**DOI:** 10.1177/00236772241262119

**Published:** 2024-10-04

**Authors:** Maya J. Bodnar, I. Joanna Makowska, Courtney T. Boyd, Catherine A. Schuppli, Daniel M. Weary

**Affiliations:** 1 UBC Animal Welfare Program, Faculty of Land and Food Systems, The University of British Columbia, Vancouver, Canada

**Keywords:** Euthanasia, conditioned place aversion, drop method, isoflurane, mice, refine, welfare

## Abstract

Isoflurane anesthesia prior to carbon dioxide euthanasia is recognized as a refinement by many guidelines. Facilities lacking access to a vaporizer can use the “drop” method, whereby liquid anesthetic is introduced into an induction chamber. Knowing the least aversive concentration of isoflurane is critical. Previous work has demonstrated that isoflurane administered with the drop method at a concentration of 5% is aversive to mice. Other work has shown that lower concentrations (1.7% to 3.7%) of isoflurane can be used to anesthetize mice with the drop method, but aversion to these concentrations has not been tested. We assessed aversion to these lower isoflurane concentrations administered with the drop method, using a conditioned place aversion (CPA) paradigm. Female C57BL/6J (OT-1) mice (*n* = 28) were randomly allocated to one of three isoflurane concentrations: 1.7%, 2.7%, and 3.7%. Mice were acclimated to a light–dark apparatus. Prior to and following dark (+ isoflurane) and light chamber conditioning sessions, mice underwent an initial and final preference assessment; the change in the duration spent within the dark chamber between the initial and final preference tests was used to calculate a CPA score. Aversion increased with increasing isoflurane concentration: from 1.7% to 2.7% to 3.7% isoflurane, mean ± SE CPA score decreased from 19.6 ± 20.1 s to –25.6 ± 23.2 s, to –116.9 ± 30.6 s (*F*_1,54_ = 15.4, *p* < 0.001). Our results suggest that, when using the drop method to administer isoflurane, concentrations between 1.7% and 2.7% can be used to minimize female mouse aversion to induction.

## Aversion de la souris à l’induction à l’isoflurane en utilisant la méthode par goutte Résumé

L’anesthésie à l’isoflurane avant l’euthanasie au dioxyde de carbone (CO_2_) est reconnue comme un raffinement par de nombreuses directives. Les installations qui n’ont pas accès à un vaporisateur peuvent utiliser la méthode « par goutte », par laquelle un anesthésique liquide est introduit dans une chambre d’induction. Il est essentiel de connaître la concentration la moins aversive d’isoflurane. Des travaux antérieurs ont démontré que l’isoflurane administré avec la méthode par goutte à une concentration de 5% était aversif pour les souris. D’autres travaux ont montré que des concentrations plus faibles (1,7–3,7%) d’isoflurane pouvaient être utilisées pour anesthésier des souris avec la méthode par goutte, mais l’aversion pour ces concentrations n’a pas été testée. Nous avons évalué l’aversion à ces concentrations plus faibles d’isoflurane administrées avec la méthode par goutte en utilisant un paradigme d’aversion du lieu conditionné (CPA). Des souris femelles C57BL/6J (OT-1) (n = 28) ont été affectées au hasard à l’une des trois concentrations d’isoflurane suivantes: 1,7%, 2,7% et 3,7%. Les souris ont été acclimatées à un appareil lumière-obscurité. Avant et après les séances de conditionnement en chambre obscure (+ isoflurane) et en chambre lumineuse, les souris ont subi une évaluation initiale et finale des préférences; le changement de la durée passée dans la chambre obscure entre les tests initiaux et finaux des préférences a été utilisé pour calculer un score CPA. L’aversion augmentait avec l’augmentation de la concentration d’isoflurane; de 1,7% à 2,7% à 3,7% d’isoflurane, le score CPA moyen ± ET diminuait de 19,6 ± 20,1 s à −25,6 ± 23,2 s, à −116,9 ± 30,6 s; *F*1,54 = 15,4, *p* < 0,001. Nos résultats suggèrent que, lors de l’utilisation de la méthode par goutte pour administrer de l’isoflurane, des concentrations comprises entre 1,7 et 2,7% peuvent être utilisées pour minimiser l’aversion des souris femelles à l’induction.

## Aversion von Mäusen gegen die Isofluran-Induktion mit der Tropfenmethode Abstract

Die Anästhesie mit Isofluran vor der Tötung mit Kohlendioxid (CO_2_) gilt in vielen Leitlinien als Verbesserung. Einrichtungen, die keinen Zugang zu einem Verdampfer haben, können die Tropfenmethode anwenden, bei der ein flüssiges Anästhetikum in eine Induktionskammer gegeben wird. Es kommt dabei wesentlich darauf an, die am wenigsten aversive Isofluran-Konzentration zu kennen. Frühere Arbeiten haben gezeigt, dass Isofluran, das mit der Tropfenmethode in einer Konzentration von 5% verabreicht wird, für Mäuse aversiv ist. Andere Untersuchungen ergaben, dass niedrigere Isofluran-Konzentrationen (1,7–3,7%) zur Betäubung von Mäusen mit der Tropfenmethode verwendet werden können, allerdings wurde die Aversion gegen diese Konzentrationen nicht getestet. Wir untersuchten die Aversion gegenüber diesen niedrigeren Isofluran-Konzentrationen, die mit der Tropfenmethode verabreicht wurden, mit einem Paradigma der konditionierten Ortsaversion (CPA). Weibliche C57BL/6J (OT-1)-Mäuse (n = 28) wurden nach dem Zufallsprinzip einer von drei Isofluran-Konzentrationen zugewiesen: 1,7%, 2,7% und 3,7%. Die Mäuse wurden an einen Hell-Dunkel-Mechanismus akklimatisiert. Vor und nach den Konditionierungsphasen in der dunklen (+ Isofluran) und der hellen Kammer wurden die Mäuse jeweils einer anfänglichen und abschließenden Präferenzbewertung unterzogen; die Veränderung der in der dunklen Kammer verbrachten Zeit zwischen den anfänglichen und abschließenden Präferenztests wurde zur Berechnung eines CPA-Scores verwendet. Die Aversion nahm mit steigender Isofluran-Konzentration zu; von 1,7% über 2,7% bis 3,7% Isofluran sank der mittlere CPA-Wert ± SE von 19,6 ± 20,1 s über −25,6 ± 23,2 s auf −116,9 ± 30,6 s; *F*1,54 = 15,4, *p* < 0,001. Unsere Ergebnisse deuten darauf hin, dass bei Verwendung der Tropfenmethode zur Verabreichung von Isofluran Konzentrationen zwischen 1,7 und 2,7% verwendet werden können, um die Aversion weiblicher Mäuse gegen die Induktion zu minimieren.

## Aversión del ratón a la inducción con isoflurano mediante el método de la gota Resumen

La anestesia con isoflurano previa a la eutanasia con dióxido de carbono (CO_2_) está reconocida como un refinamiento por muchas directrices. Los centros que no tengan acceso a un vaporizador pueden utilizar el método de la “gota”, por el que se introduce anestésico líquido en una cámara de inducción. Conocer la concentración menos aversiva de isoflurano es primordial. Trabajos anteriores demostraron que el isoflurano administrado con el método de la gota a una concentración del 5% es aversivo para los ratones. Otros estudios han demostrado que pueden utilizarse concentraciones más bajas (1,7–3,7%) de isoflurano para anestesiar ratones con el método de la gota pero no se ha comprobado la aversión a estas concentraciones. Evaluamos la aversión a estas concentraciones más bajas de isoflurano administradas con el método de la gota, utilizando un paradigma de aversión condicionada al lugar (CPA). Los ratones hembra C57BL/6J (OT-1) (n = 28) fueron asignados aleatoriamente a una de las tres concentraciones de isoflurano: 1,7%, 2,7%, y 3,7%. Los ratones fueron aclimatados a un aparato de luz-oscuridad. Antes y después de las sesiones de acondicionamiento en cámara oscura (+ isoflurano) y luminosa, los ratones se sometieron a una evaluación inicial y final de las preferencias; el cambio en la duración de permanencia en la cámara oscura entre las pruebas de preferencia inicial y final se utilizó para calcular una puntuación CPA. La aversión aumentó con el incremento de la concentración de isoflurano; del 1,7% al 2,7% y al 3,7% de isoflurano, la puntuación CPA media ± SE disminuyó de 19,6 ± 20,1 s a −25,6 ± 23,2 s, a −116,9 ± 30,6 s; *F*1,54 = 15,4, *p* < 0,001. Nuestros resultados sugieren que, cuando se utiliza el método de la gota para administrar isoflurano, pueden emplearse concentraciones entre el 1,7 y el 2,7% para minimizar la aversión de las hembras de ratón a la inducción.

## Introduction

Laboratory mice are often euthanized with carbon dioxide (CO_2_), a method known to be aversive.^1–3^ Euthanasia guidelines often encourage methods to help minimize pain and distress, such as the use of inhalant anesthetics before exposure to CO_2_.^4,5^ Isoflurane is commonly used to render mice unconscious prior to CO_2_ exposure, and is valued for the rapid onset of anesthesia and high safety profile.^6,7^ Isoflurane exposure is known to be aversive, but less so than CO_2_.^3,8,9^ Isoflurane is typically administered using a vaporizer and carrier gas (e.g., oxygen), but access to a vaporizer can be limiting, preventing some from adopting the method.^10^

An alternative to vaporizers is the “drop” method, whereby a fixed quantity of liquid anesthetic is introduced into a sealed chamber. Once introduced, isoflurane volatilizes and allows for rapid induction. This method is commonly used when anesthetizing wildlife in field research (e.g., Parker et al.^11^; Potvin et al.^12^) and can also be used to anesthetize laboratory mice.^13–15^ Previous work has shown that isoflurane delivered via the drop method to achieve a concentration of 5% is aversive to mice.^14^ Recently, we demonstrated that mice can be successfully anesthetized via the drop method using lower isoflurane concentrations (ranging from 1.7% to 3.7%^15^), which are thought to be less aversive,^3^ but to date no work has assessed aversion to these concentrations administered via the drop method. Understanding aversion at these lower concentrations is critical for refining euthanasia protocols.

Conditioned place aversion (CPA) paradigms are commonly used to assess the aversive properties of drugs.^16^ This paradigm involves conditioning animals to associate distinctive characteristics of an enclosure with exposure to a stimulus. Before conditioning, mice were tested in a light–dark apparatus to determine their individual, pre-existing preference for either a dark or brightly lit chamber. Mice were then exposed to a number of conditioning sessions in which they were exposed to isoflurane in the dark chamber, and on alternating days, to the brightly lit chamber that was isoflurane free. After multiple conditioning sessions, the animals were again placed in the two-chamber arena in the absence of any isoflurane. A reduction in the amount of time they spent in that isoflurane-paired dark chamber, compared to their pre-conditioning baseline, was used as a measure of aversion (following Watanabe et al.^17^). We hypothesized that female mouse aversion to isoflurane administered via the drop method would increase with increasing concentration, as demonstrated by less time spent in the dark chamber associated with isoflurane.

## Methods

All animal procedures were approved by The University of British Columbia’s Animal Care Committee (protocol A20-0269).

### Animals and housing

We received 30 experimentally naïve surplus female C57BL/6J (OT-1) mice slated for euthanasia. Mice were originally purchased from a commercial vendor (The Jackson Laboratory, Bar Harbor, USA). These transgenic mice have a genetic modification to express the OT-1 T cell receptor. This strain is commonly used in immunological research, and the genetic modification was not expected to have any impact on anesthetic sensitivity. All mice were naïve to isoflurane exposure.

All mice were specific-pathogen-free. Daily health assessments were performed by facility animal care technicians, and the experimenters regularly observed for any signs of illness throughout the experimental period. One mouse was observed to have anophthalmia and was therefore excluded from participating in the study. Prior to beginning habituation trials, another mouse was discovered deceased in her cage, with no apparent cause of death. As a result, the final sample size for this study was 28 mice. Our sample size (*n* = 9 or *n* = 10 per treatment) was based on that used in previous studies assessing anesthesia in rodents (*n*s = 8 to 13).^13–15^ Mice were transferred to our experimental protocol already marked with ear notches, which were used to identify individual mice within the cages. At the time of testing, mice were 13–21 weeks of age and weighed (mean ± SD) 26.2 ± 4.5 g at the beginning of testing and 29.9 ± 5.1 g at the end of testing. Mice were housed in groups of 4–5, consistent with their previous housing.

Upon arrival at our laboratory, mice were transferred to larger open top rat cages measuring 39.4 cm long, 30.0 cm wide, and 19.4 cm high (Allentown, LLC, Allentown, USA). Cages contained aspen chip bedding (Jamieson’s Pet Food Distributors Ltd, Delta, Canada), nesting material (Enviropak nesting material, Datesand, Stockport UK; cotton nestlet, Ancare, Bellmore, USA), and three red polycarbonate huts (Bio-Serv, Flemington, USA). *Ad libitum* food (Lab Diet Rodent Chow 2918) and reverse osmosis tap water were provided. Cages were changed every other week by the primary experimenter and mice were handled with an overturned hut or clear handling tunnel during cage changes. The animal room was kept on a reversed 12 h:12 h light cycle, with lights off from 09:00 to 21:00. Mice were housed at a (mean ± SD) room temperature of 21.4 ± 0.1°C and a relative humidity of 49.5 ± 0.05%.

### Experimental design

Individual mice were randomly allocated to one of the three isoflurane treatment concentrations: 1.7% (*n* = 10), 2.7% (*n* = 9), and 3.7% (*n* = 9) using a Latin-square design. To ensure timely completion of daily conditioning, mice were divided into two cohorts (*n* = 15 and *n* = 13), with testing of the second cohort beginning immediately after the completion of testing the first cohort. Testing order within cages was assigned randomly in advance and was maintained throughout the experiment.

### Apparatus

**Figure 1.**
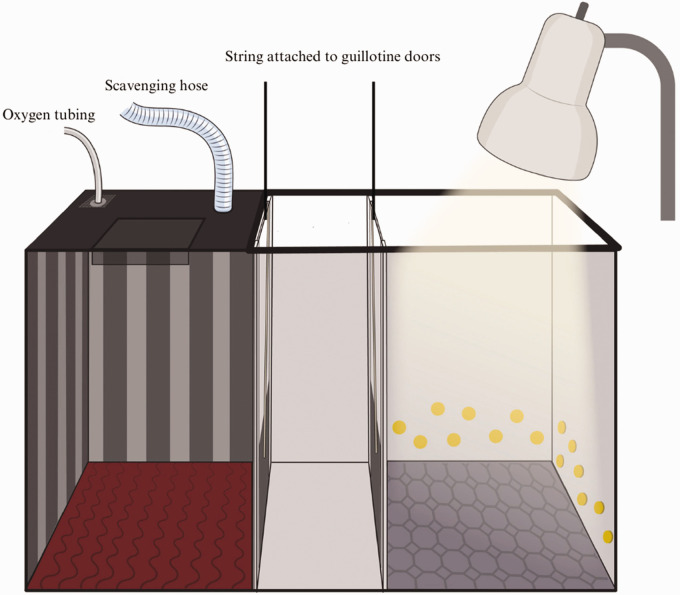
Illustration of our experimental CPA apparatus consisting of three connected chambers: dark, middle, and light (from left to right in the illustration) with distinct somatosensory cues. Guillotine doors are located along either side of the middle chamber, separating this chamber from the dark and light chambers. Two holes on the lid of the dark chamber are for a scavenging hose and to flush the chamber with oxygen between trials. The oxygen tubing was sealed during trials. The cotton pad and plastic mesh are located directly below the attached oxygen tubing. The clamped oxygen tubing was placed in the hole following the administration of isoflurane (Baxter Corporation, Mississauga, Canada) onto the cotton pad via a syringe.

A 3-chamber light–dark box (61.5 cm long, 20 cm wide, and 35.5 cm high) was used as the test apparatus (Figure 1). The light chamber was lit to 700 lux by a desk lamp with a 60 W LED bulb, consistent with previous work using a light–dark paradigm to assess mouse aversion to isoflurane.^14^ The light and dark chambers contained various somatosensory cues to differentiate the chambers: the dark chamber contained red silicone flooring with ridges and a striped black and white wall, and the light chamber contained textured blue foam flooring with yellow plastic buttons attached to the walls.

Two lids were made of clear acrylic sheet to fit over the dark chamber (21 × 26 cm) and the middle and light chambers (21 × 37 cm), respectively. Foam tape was secured along the underside perimeter of each lid and the lid covering the dark chamber was covered by an opaque black sheet to block surrounding light. A square portion of the opaque black sheet was cut out in the lid to create a viewing flap, which could be opened and closed during trials to observe the mouse. Two 1.5-cm diameter holes were drilled into the lid of the dark chamber. One hole allowed for the placement of a hose connected to a waste scavenging system, while the other served a dual role: facilitating the administration of isoflurane with a 5 mL syringe, and to accommodate tubing to flush oxygen between trials. Directly below this second hole, a square piece of plastic mesh was secured against the underside of the lid with an open slot to slide in a compressed cotton pad (Quo Beauty, Toronto, Canada) that absorbed the isoflurane dispensed from the syringe. Upon administering isoflurane onto the cotton pad, clamped oxygen tubing was placed in the hole to prevent any dissipation of isoflurane. This tubing was unclamped between conditioning sessions to flush the chamber with oxygen. Between conditioning sessions, the apparatus lid was left ajar to allow for any remaining gases to dissipate. Two guillotine doors made of clear acrylic sheets (7.5 × 11 cm) were placed in the doorways connecting the chambers to the middle chamber. The door to the dark chamber was wrapped in black electrical tape to block light. String was attached to both doors, which were accessed through 1-mm holes drilled in the lid of the light and middle chambers. The experimenter controlled the free ends of the strings, allowing for manual control of the guillotine doors.

The volume of isoflurane was calculated to achieve the desired concentrations of 1.7%, 2.7%, and 3.7% using the ideal gas law (PV = nRT; where P is pressure, V is volume, n is number of moles of isoflurane, R (8.314) is the universal gas constant, and T is temperature) with 21°C as the room temperature, resulting in volumes of isoflurane of 2.3, 3.7, and 5.1 mL.

### Experimental procedure

All habituation and test trials took place in a room lit only with red light. Procedures were performed on a backdraft table to scavenge waste gas. The mice were individually transported to and from the procedure room in an empty Allentown rat cage. They were transferred into and out of cages using a handling tunnel or overturned hut. Following the trial, the mice were transported back to the animal room and kept in a holding cage (another empty Allentown rat cage). The mice were returned to their home cage after all the mice from the same cage had completed testing. After each trial, the CPA apparatus and recovery cage were cleaned with a damp, unscented soapy cloth and rinsed with a separate damp cloth. Between cages, the transport cage was washed with unscented soap and rinsed with water.

#### Habituation

The mice were habituated to the procedure room and apparatus in 5-min sessions over six consecutive days. During habituation sessions, the mouse was transported to the procedure room and placed into the middle chamber of the CPA apparatus using a handling tunnel. Upon securing the apparatus lids, the light and dark chamber guillotine doors were raised, and the mouse was allowed to explore the apparatus for 3 min. After 3 min of free exploration (±10 s to ensure the mouse was confined to alternate sides during each session), each mouse was locked in either the light or dark chamber of the apparatus for 2 min, alternating sides across habituation days.

Following the apparatus habituation sessions, the mice were habituated to the recovery conditions that would be experienced after conditioning. For this, the mouse was transferred into a mouse cage measuring 29.7 cm long, 18.8 cm wide, and 12.7 cm high (Allentown, LLC, Allentown, USA), which was lined with paper towel and contained a hut. The cage was pre-warmed with a heating pad (Sunbeam Products Inc., Boca Raton, United States) placed under one half of the recovery cage, with the temperature set to 35°C to 40°C. The lid of the recovery cage was propped open to allow for the placement of tubing with oxygen flowing at 2 L/min. After 60 s, the mouse was transferred back into her home cage.

#### Initial preference assessment

After six habituation sessions to the CPA apparatus, the mice underwent an initial preference assessment to determine baseline preference for the dark chamber. During the assessment, the guillotine doors were removed. The mice were individually transferred into the middle chamber of the apparatus and given 15 min to explore.

#### Conditioning sessions

Following initial preference testing, the mice were conditioned to the dark chamber (+ isoflurane) and light chamber of the apparatus. They were exposed to the dark (+ isoflurane) and light chamber on alternating conditioning sessions (i.e., mice underwent dark chamber conditioning on one day, and light chamber conditioning the next). This alternating schedule continued for 12 consecutive days, excluding 2-day weekend breaks, totaling 6 dark (+ isoflurane) and 6 light chamber conditioning sessions.

To increase mouse agency during the sessions and assess whether conditioning can occur in fewer than six sessions, the mouse could choose whether to enter the dark chamber during conditioning sessions. The mouse was placed in the middle chamber and the guillotine doors were opened to allow exploration. Upon entering the dark chamber, the guillotine door was closed, and the isoflurane was administered. The experimenter opened the viewing flap of the dark chamber lid every 20 s to note recumbency (defined as the first instance where the mouse was lying down with her head on the floor with visible loss of muscle tone). Isoflurane-conditioning trials ended after 2 min or at recumbency, whichever occurred first. After their trial, the mouse was placed in the pre-warmed recovery cage for 60 s.

During light conditioning sessions, the mouse was placed directly into the light chamber with the guillotine door already closed and was confined to this side for 2 min. To maintain similar conditions following the dark (+ isoflurane) conditioning days, mice were also placed in the pre-warmed recovery cage following each conditioning session.

Using an aversion–avoidance paradigm, Boulanger Bertolus et al.^18^ ended the trials when the rats left the bottom cage associated with isoflurane for more than 90 s. However, mice are known to exhibit higher frequencies of entries and exits than rats.^19^ On this basis, we considered mice to have met conditioning criteria if they chose not to enter the dark chamber within 45 s for two consecutive trials.

#### Final preference assessment

Final preference assessments were conducted once the mice had reached conditioning criteria or had completed all 12 conditioning sessions (six light and six dark chamber), whichever occurred first.

### Video and statistical analysis

All trials were video recorded (Canon VIXIA HF W10, Tokyo, Japan) and scored using Behavioral Observation Research Interactive Software (BORIS, version v. 7.13.6) by an observer blind to treatment. All mice (*n* = 28) were included in the analysis. Preference assessment video observations were scored for duration spent in the light and dark chambers. Dark (+ isoflurane) conditioning sessions were scored for latency to enter the dark (+ isoflurane) chamber, latency to ataxia (defined as the onset of observable loss of motor function or balance), and latency to recumbency. Twenty-five percent of the videos were scored by a second blinded observer to calculate interobserver reliability for the latency to enter the dark (+ isoflurane) chamber during conditioning days, as well as the duration spent in the same chamber during preference assessments. The Pearson correlation coefficient was calculated as *r* = 0.99 for both measures. A CPA score was calculated by subtracting the time spent in the isoflurane-paired compartment pre-conditioning from post-conditioning.

**Figure 2.**
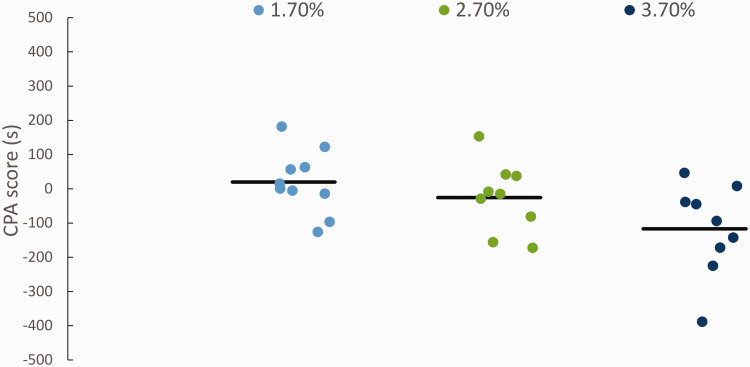
CPA score (s) declined with increasing concentration of isoflurane (*p* < 0.001). Black bars show treatment means, and individual points show the values for each mouse (*n*s = 10, 9, and 9 for the 1.7%, 2.7%, and 3.7% treatments, respectively). Points are jittered horizontally to better visualize overlapping points.

**Figure 3.**
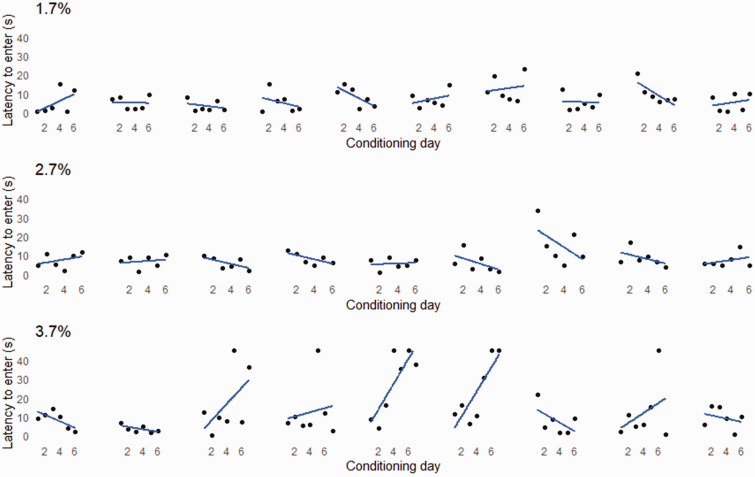
Changes in latency to enter the dark (+ isoflurane) chamber across conditioning sessions. Each plot represents an individual mouse (*n*s = 10, 9, and 9 mice in the 1.7%, 2.7%, and 3.7% treatments). The maximum possible latency to enter the dark (+ isoflurane) chamber was 45 s.

Statistical analysis was carried out using SAS OnDemand for Academics (release 3.81, SAS Institute Inc.) A general linear mixed model was used to test the effect of isoflurane concentration on CPA score. All models specified cage as a random factor. Figure 2 was generated in Microsoft Excel (version 2308), and Figure 3 was generated in R studio (R version 4.0.1) using the ggplot2 package.

## Results and discussion

This study investigated mouse aversion to isoflurane administered via the drop method at concentrations of 1.7%, 2.7%, and 3.7%. During the initial preference assessment, the mice spent an average of 372.6 ± 16.2 s in the dark (+ isoflurane) chamber and 305.8 s ± 16.1 s in the light chamber of the apparatus. Consistent with the innate unconditioned preference for dark versus bright environments in mice, we had expected almost all mice to spend more time in the dark chamber during the initial (baseline) preference assessment; however, 8 out of 28 mice spent more time in the light chamber. While acclimation to the light–dark apparatus typically produces aversion to the light chamber,^20^ some mice in the present study may have been attracted to specific features of the light chamber that differed from the dark chamber, such as the plastic buttons attached to the walls.

All mice underwent six isoflurane conditioning sessions, except for one mouse in the 3.7% treatment group who reached conditioning criteria in five sessions. Four additional mice in the same treatment chose not to enter the dark (+ isoflurane) chamber once and were thus exposed to an additional dark (+ isoflurane) conditioning session to reach a total of six sessions.

Six forced-exposure sessions are suggested as the standard needed for mice to form an association between the environment and agent.^1^ However, we sought to refine this standard test by providing the mice with agency to enter the dark (+ isoflurane) chamber; therefore, they were given the opportunity to reach conditioning criteria in fewer than six conditioning sessions. Five mice in the 3.7% treatment chose not to enter the dark (+ isoflurane) chamber at least once; this result is consistent with aversion resulting from previous exposures.

The mice varied in CPA score, particularly in the 3.7% treatment (Figure 2). In this treatment, scores ranged from +46.5 to –388.5 s: increasingly negative scores indicate heightened aversion. Responses to behavioral assessments, such as the light–dark paradigm, can vary throughout the female estrous cycle.^21^ Our study only used female mice, which may have contributed to the variability. Further, individual differences in aversion to CO_2_ in rats have been attributed to differences in individual personality traits^22^; similarly, individual variability in aversion to isoflurane may be due to differences in the personality traits of the mice.

As isoflurane concentration increased from 1.7% to 2.7%, and finally to 3.7%, mean ± SE CPA score decreased from 19.6 ± 20.1 s (*n* = 10) to –25.6 ± 23.2 s (*n* = 9) and finally to –116.9 ± 30.6 s (*n* = 9); *F*_1,54_ = 15.4, *p* < 0.001 (Figure 2). This dose response indicates that aversion became more pronounced with increasing concentrations of isoflurane, a result that is consistent with previous work using the drop method that found that isoflurane was aversive to mice at a concentration of 5%.^14^

Most mice became recumbent during at least one isoflurane conditioning session, and all mice in the 3.7% treatment became recumbent at least once. Across all isoflurane concentrations, mice who became recumbent had lower CPA scores than mice who did not (–78.0 ± 26.8 s vs. 44.0 ± 28.2 s). This result suggests that experiences preceding loss of consciousness, including recumbency, heighten aversion. We suggest future research includes the use infrared or low-light cameras in the dark chamber to more precisely assess times to insensibility and how this may affect aversion.

We expected that latency to enter the dark chamber would increase across isoflurane conditioning sessions, but our results showed little evidence of change in latency to enter the dark chamber across sessions, especially for the 1.7% and 2.7% treatments (Figure 3). Some mice in the 3.7% treatment showed a pronounced increase in their latency to enter the dark chamber during later conditioning sessions, driving the lower mean CPA score for this treatment. Previous work has reported that repeated exposure to volatile anesthetics in rodents is more aversive compared to the initial exposure,^9,14,18^ but each of these studies used higher concentrations than those assessed in the current study. We suspect this learned aversion may be related to the feeling of experiencing an altered state of consciousness. For example, rats are known to avoid stimuli that result in a state change,^23,24^ suggesting a potential fear response.^25^ In the present study, the mice exposed to 3.7% isoflurane became recumbent more frequently and rapidly compared to those exposed to lower concentrations of isoflurane, potentially contributing to heightened aversion.

The concentrations reported in this study (1.7%, 2.7%, 3.7%) indicate the final concentrations once all the isoflurane had volatilized. However, users should be aware that the rate of increase (i.e., how quickly the final concentration will be achieved) will depend on several factors, including the size and characteristics of the cotton pad onto which liquid isoflurane is poured (i.e, the evaporation surface area), and the temperature within the chamber. The positioning of the liquid isoflurane (e.g., under the lid or on the cage floor) will also affect the pattern of dispersion within the cage; isoflurane is denser than air and thus will settle on the bottom of the induction chamber if undisturbed.^26^ The rate of dispersion most likely affects mouse aversion to induction, so the results here hold true to the apparatus specifications used in this study. Future research should explore how varying these factors influences induction and aversion to isoflurane in mice, ideally using a gas analyzer to document variation in anesthetic concentration within the chamber.

When using the drop method, using less liquid isoflurane (i.e., to achieve lower isoflurane concentrations) not only refines mouse induction with this agent, but also reduces the environmental impact of halogenated anesthetics. Moreover, compared to the use of a vaporizer and carrier gas, the drop method allows for very rapid volatilization of isoflurane, resulting in further reductions in use. There has been growing concern surrounding the use of general anesthetics on greenhouse gas emissions and their subsequent contribution to global warming. Notably, isoflurane has a relatively long atmospheric lifetime and consequent global warming potential.^27,28^ Thus, we conclude that the drop method could also be considered an environmental refinement.

As with any use of potential toxins in laboratories, use of the drop method requires careful consideration of the safety of personnel. For example, to reduce the risk of exposure to isoflurane, all work should be conducted under a biological safety cabinet or with adequate backdraft and scavenging of waste gases.

## Conclusion

Female mouse aversion to isoflurane delivered using the drop method increases with isoflurane concentration; there was minimal evidence of aversion at lower concentrations of 1.7% and 2.7% compared to 3.7%. The use of the drop method at these lower concentrations provides an opportunity to refine mouse isoflurane anesthesia prior to CO_2_ euthanasia, even in laboratories that lack access to a gas vaporizer.

## Data Availability

All data and code used for the statistical analysis are available on the UBC dataverse (https://doi.org/10.5683/SP3/VIURRC).
